# Longitudinal policy surveillance of state obesity legislation in California, 1999–2020

**DOI:** 10.1186/s12889-024-20557-y

**Published:** 2024-11-06

**Authors:** Denise D. Payán, Alec M. Chan-Golston, Kesia K. Garibay, Corbin Farias

**Affiliations:** 1 Department of Health, Society and Behavior, Joe C. Wen School of Population & Public Health, University of California, Irvine, 856 Health Sciences Quad, Irvine, CA 92697-3957 USA; 2 Department of Public Health, School of Social Sciences, Humanities, and Arts, University of California, Merced, 5200 North Lake Rd, Merced, CA 95343 USA

**Keywords:** Obesity, Policy, Surveillance, Law, Nutrition, Physical activity

## Abstract

**Background:**

Obesity rates among children and adults continue to accelerate in the U.S., particularly among marginalized and low-income populations. Obesity prevention and reduction policies can significantly impact population health by improving environmental conditions and increasing access to health-promoting resources. Limited research has been conducted to examine state obesity policy change over time. The primary aim of this study is to examine legislative approaches used to prevent and reduce obesity in the state of California (U.S.).

**Methods:**

We used quantitative policy surveillance methods to develop a state database of obesity-related legislation (bills, resolutions) introduced in California’s legislature between 1999 and 2020. Descriptive statistics were used to examine trends of introduced and enacted policy by legislative and policy characteristics. Chi-square tests were used to determine differences in characteristics between enacted and non-enacted legislation. Legislative session and policy characteristics found to be associated with enactment were used to predict adoption in a logistic regression.

**Results:**

A total of 284 obesity-related bills and resolutions were introduced in California’s legislature between 1999 and 2020 with a peak of 43 in 2005–2006. On average, 25.8 bills and resolutions were introduced each 2-year legislative cycle. Findings indicate that (a) children and schools were the most frequently specified population and setting; (b) the most common policy topics were nutrition (45%) and physical activity (33%); and (c) only 15% of legislation mentioned race/ethnicity. Overall, 24.9% of bills were enacted compared to 82.1% of resolutions adopted. Legislation to raise awareness about obesity had 5.4 times the odds of being passed compared to other topics. Yet this difference was not statistically significant in a sensitivity analysis when we excluded resolutions.

**Conclusions:**

This database can be leveraged to advance our knowledge of effective and equitable policy instruments to prevent and reduce obesity. Results reveal important policy elements that may impact legislative success, including policy topic, and contribute to a nascent evidence base for public health law research, legal epidemiology, and practice. Future work should investigate the role of policy effectiveness research and evidence on legislative policymaking.

**Supplementary Information:**

The online version contains supplementary material available at 10.1186/s12889-024-20557-y.

## Background

It is essential to examine policies to address and prevent obesity in order to comprehensively assess governmental efforts to reduce non-communicable disease rates. In terms of health impacts, obesity increases risk of cardiovascular disease, type 2 diabetes, stroke, osteoarthritis, respiratory conditions (including sleep apnea), various cancers [1], and depression [2], and contributes to several leading causes of death in the United States (U.S.). However, efforts to surveil and monitor obesity policy trends are limited compared to surveillance programs that track and collect individual-level health behavior and outcomes data [3].

In the U.S., the obesity epidemic was elevated to the forefront of the nation’s health policy agenda at the turn of the 21st century due to mounting evidence of the associated health effects, cost, and disproportionate impact on marginalized and low-income populations [4]. States and local municipalities responded with policies and programs to improve dietary and physical activity behavior—serving as natural laboratories of policy experimentation and innovation. Despite this policy surge, little is known about state obesity policy change over time, the types of policy topics that have been prioritized by policymakers, and how policy-level factors might influence enactment. Some of these policies aim to address market failures, including imperfect information about food consumed (i.e., nutrition and calorie labeling) or negative external costs from health burdens (i.e., taxing or subsidizing foods or behaviors) [5]. While obesity prevention and reduction policies can impact population health by improving environmental conditions and increasing access to health-promoting resources, policy surveillance research is needed to build an evidence base for policy evaluation and to support public health law research and legal epidemiology [6, 7].

State legislation is a potentially critical policy data repository, which includes interventions and funding for programs to address the obesity epidemic. Related articles have examined patterns and predictors of legislation targeting obesity [8–10], childhood obesity [11–14], adult obesity [15], and physical activity [16] across states. Prior studies found obesity legislation targeting schools, health and nutrition content, and physical activity were the most frequently proposed and enacted types of bills [10, 13, 15].

Investigating obesity policy trends over time *within* a state can reveal important details about policy instruments used, topic patterns, and intervention effectiveness. Limitations with existing work include a focus on a particular behavioral determinant (e.g., physical activity), narrow target audience (e.g., children), or brief study period (i.e., 2–3 years). Policy change studies using decades of data are more rigorous than cross-sectional studies [17] covering short periods [18].

California has served as a laboratory for obesity policy—enacting more obesity legislation than other states [10–13, 15]. Also recognized as an early adopter of new legislation, California was the first to adopt a mandatory menu labeling law in 2008 [19] and a healthy kids’ meal law in 2018 [20]—both were subsequently adopted or are being considered in multiple states.

The primary objective of this study is to use policy surveillance methods to produce a longitudinal state database of obesity-related legislation introduced in California’s legislature between 1999 and 2020. We also analyze change trends by policy topic and content.

## Methods

### Data Collection

We use policy surveillance, a process for measuring the law [17] defined as the “systematic, scientific collection and analysis of laws of public health significance” [7], to develop a state obesity policy database for California. Policy surveillance is a method intended to systematically identify and analyze laws (and/or other types of policies) using inclusion and exclusion criteria, and a coding scheme to measure and track how these laws change over time [7, 21].

We systematically searched for obesity prevention and reduction legislation introduced in California’s bicameral legislature between January 1, 1999 – December 31, 2020. As bills could be introduced during a 2-year legislative session, the study period covered 11 legislative sessions. The search process focused on identifying proposed legislation with search terms “obesity”, “obese”, or “overweight” using the state’s legislative information website [22]. Search terms reflect the problematized health issue of concern from the perspective of policy stakeholders since the policy making cycle is often theorized as starting with the agenda setting process where the problem is identified prior to policy formulation or adoption [23].

Legislation included bills and resolutions. For clarification, a bill is a proposed new law or amendment to an existing law presented to the legislature for consideration, while resolutions are a formal expression of the will, opinion, or direction of one or both chambers of the legislature on a matter of public interest, and do not require gubernatorial action. A detailed description with definitions and a diagram of the state of California’s legislative process is available online [24].

Two research assistants were trained to independently conduct the initial search (1999–2019) in early 2020. A graduate student conducted a follow-up search using the same criteria to include bills and resolutions introduced through December 31, 2020 and to update relevant legislative materials through the end of the legislative term. These searches yielded *n* = 354 bills or resolutions. We conducted validation checks by searching for other terms, including health behaviors (e.g., “physical activity”), to compare search output. We supplemented the database by comparing the output with California-specific legislative data in two existing policy databases [25, 26]. We included *n* = 4 relevant bills from these existing databases that were not identified in our search process.

At least two research staff reviewed each title and legislative summary to assess for relevance, leading to the exclusion of *n* = 42 focused on non-health topics (e.g., overweight trucks/cargo). Assistants downloaded related documents from the state’s legislative website, including the text, votes, history, bill analysis, amendments, and status. A third research assistant reviewed the entire content of the remaining documents, leading to the exclusion of *n* = 32 that aimed to protect the industry from civil liability lawsuits (*n* = 3), were solely budget bills (*n* = 6), or lacked any obesity prevention or reduction mechanism (*n* = 23). *N* = 284 bills and resolutions were included in the final analyses. Supplement Figure S1 details the systematic search process.

The lead author used a primarily deductive approach to develop a draft codebook to reflect themes identified a priori from policy surveillance and state obesity policy literature [11–13, 15, 16, 25–30]. Two research assistants pilot tested the codebook by independently coding a subset of proposed legislation for comparison and to identify issues, including the need to expand or clarify a definition or measure. A third assistant used the final codebook to extract pertinent policy data from legislative documents to input in an Excel spreadsheet. Coding questions or issues were addressed in biweekly team meetings or by email. The lead author conducted quality assurance assessments to identify and address missing data issues, randomly selected coded content for comparison, and reviewed response options to ensure they were within appropriate and expected parameters.

### Measures

The codebook had four categories and 32 variables, including policy details (11 variables), policy topics (10), policy content (6), and policy instruments (5). The full codebook with variable names, definitions, code/values, and references is available online [31].

Key variables for the analysis consisted of: legislation type (bill, resolution), originating legislative chamber (Assembly, Senate), legislative success, veto status, session years, policy topic (physical activity, nutrition/diet, education, healthcare, awareness, environmental health, domestic or child abuse, housing instability/insecurity), target age (child, adult, older adult, none), target setting (school or early childhood, healthcare, restaurant/food facility, employment/worksite, other, none), and whether a specific race/ethnic group was mentioned. Of note, a bill could include multiple components and be coded for more than one topic. For example, a bill aimed at improving nutrition education would be coded as both nutrition/diet and education.

### Statistical analysis

Descriptive statistics were used to examine trends of introduced and enacted policy by legislative and policy characteristics. Chi-square tests were used to determine differences in characteristics between enacted and non-enacted legislation. Legislative session and characteristics found to be associated with enactment were used to predict adoption in a logistic regression. Statistical analyses were replicated including only bills—and excluding resolutions—as a sensitivity analysis. Cluster standard errors by legislature were included to prevent potential heteroscedasticity. P-values < 0.05 were considered statistically significant. All analyses were implemented using STATA 18.0 and data visualizations were constructed using R 4.2.3.

## Results

A total of 284 bills and resolutions (189 bills, 95 resolutions) were introduced in California’s bicameral state legislature between 1999 and 2020 with a peak of 43 bills and resolutions in 2005–2006. An average of 25.8 bills and resolutions were introduced each two-year legislative cycle (SD = 10.4), with an average of 15.6 (SD = 6.9) in the Assembly and 10.3 (SD = 4.8) in the Senate.

Overall, 44% of introduced obesity bills and resolutions (*n* = 125) were successfully enacted or adopted. Only about a quarter (24.9% or *n* = 47) of the bills were enacted into legislation compared to 82.1% (*n* = 78) of resolutions that were adopted. Among introduced bills, 11.1% (*n* = 21) were passed by the state legislature and vetoed by the governor – a majority of these (*n* = 12) were vetoed by Republican Governor Arnold Schwarzenegger.

## Enactment status by legislative Chamber and Session

Table 1 presents frequencies for introduced, not enacted, and enacted bills and resolutions by legislative chamber and session. A majority of introduced bills and resolutions, 60.2% (*n* = 171), originated in the Assembly, which had a higher share of enacted bills compared to the Senate (60% vs. 40%). Rates were similar to each chamber’s percentage of non-enacted bills.

Between 1999 and 2006, there was an increased number of bills and resolutions submitted in both legislative chambers with a steep decline in 2009–2010. There were no significant differences in the rate of enactment/adoption by legislative session (*p* = 0.597). Nor was there a difference among legislation introduced in either the Assembly (*p* = 0.284) or Senate (*p* = 0.377).

**Table 1:** Legislative and policy characteristics of introduced, not enacted, and enacted bills and resolutions to address/prevent obesity in California (*N* = 284), 1999–2020

	Bills Introduced (*N* = 284) *n* (%)	Not Enacted (*N* = 159) *n* (%)	Enacted (*N* = 125) *n* (%)
**Legislative Chamber- Origination**			
Assembly	171 (60.2)	96 (60.4)	75 (60.0)
Senate	113 (39.8)	63 (39.6)	50 (40.0)
**Legislative Year**			
1999–2000	7 (2.5)	4 (2.5)	3 (2.4)
2001–2002	14 (4.9)	8 (5.0)	6 (4.8)
2003–2004	24 (8.5)	11 (6.9)	13 (10.4)
2005–2006	43 (15.1)	24 (15.1)	19 (15.2)
2007–2008	38 (13.4)	22 (13.8)	16 (12.8)
2009–2010	19 (6.7)	12 (7.6)	7 (5.6)
2011–2012	34 (12.0)	19 (12.0)	15 (12.0)
2013–2014	25 (8.8)	12 (7.6)	13 (10.4)
2015–2016	22 (7.8)	14 (8.8)	8 (6.4)
2017–2018	29 (10.2)	12 (7.6)	17 (13.6)
2019–2020	29 (10.2)	21 (13.2)	8 (6.4)
**Target Population** ^1^			
Infants (< 2 years of age)	8 (2.8)	3 (1.9)	5 (4.0)
Children (2–17 years)	101 (35.6)	56 (35.2)	45 (36.0)
Adults (18–64 years)	5 (1.8)	4 (2.5)	1 (0.8)
Older adults (65 years+)	1 (0.4)	1 (0.6)	0 (0.0)
Other	3 (1.1)	2 (1.3)	1 (0.8)
None specified	166 (58.5)	93 (58.5)	73 (58.4)
**Race/ethnicity mentioned**	42 (14.8)	24 (15.1)	18 (14.4)
**Target Setting**			
School setting/early childcare facility	82 (28.9)	54 (34.0)	28 (22.4)
Health care organization	8 (2.8)	4 (2.5)	4 (3.2)
Restaurant or food facility (excluding school cafeterias)	5 (1.8)	2 (1.3)	3 (2.4)
Employment sites or worksite	5 (1.8)	4 (2.5)	1 (0.8)
Other	13 (4.6)	8 (5.0)	5 (4.0)
None specified	171 (60.2)	87 (54.7)	84 (67.2)
**Policy Topic** ^2^			
Nutrition/diet	129 (45.4)	74 (46.5)	55 (44.0)
Physical activity	93 (32.8)	48 (30.2)	45 (36.0)
Awareness	91 (32.0)	26 (16.4)	65 (52.0)***
Education	73 (25.7)	46 (28.9)	27 (21.6)
Health care	44 (15.5)	30 (18.9)	14 (11.2)
Environmental health	29 (6.7)	15 (9.4)	4 (3.2)*
Abuse	11 (3.9)	4 (2.5)	7 (5.6)
Housing insecurity	3 (1.1)	3 (1.9)	0 (0.0)

^1^Bill specifies a target age or group (i.e., child/children)

^2^Topics were not mutually exclusive

*p < 0.05, ** *p* < 0.01, *** *p* < 0.001

## Policy characteristics and trends

Table 1 presents frequencies for introduced, not enacted, and enacted bills and resolutions by policy characteristic, including target population, whether race/ethnicity was mentioned, target setting, and policy topic.

Among introduced bills and resolutions, over a third (36%) of introduced bills and resolutions focused on children as their target population, while 59% did not mention an age group. Similarly, 29% specified a school or early childhood setting while 11% specified another type of setting (i.e., health care organization, restaurant/food facility, worksite, other). A high percentage (60.2%) did not mention a specific target setting for implementation. Only 15% (*n* = 42) mentioned race/ethnicity in the context of racial/ethnic health disparities. Among these covariates, there were no significant differences by enactment status.

45% of introduced bills and resolutions were related to nutrition, 33% to physical activity, 32% to awareness, 26% to education, and 16% to health care. Remaining bills and resolutions focused on miscellaneous topics, including environmental health, abuse, and housing insecurity. There were no significant differences in legislation rates by enactment status, except for awareness and environmental health policy topics (see Table 1 and Table S3). More than half (52%) of those enacted focused on raising awareness, while only 16% of legislation not enacted focused on awareness. Conversely, 3% of enacted bills and resolutions focused on environmental health, while 9% not enacted focused on environmental health. When stratified analyses were conducted by legislative chamber, these statistically significant differences for awareness and environmental health policy topics were consistent for enacted Assembly legislation, but not Senate legislation. Legislation focused on the topic of abuse was also found to be significantly different for enacted legislation in the Assembly (see Supplementary materials – Tables S1 and S2). When we conducted a sensitivity analysis and excluded resolutions, we found no significant differences were detected by enactment status (results available in Table S3).

**Fig. 1:**
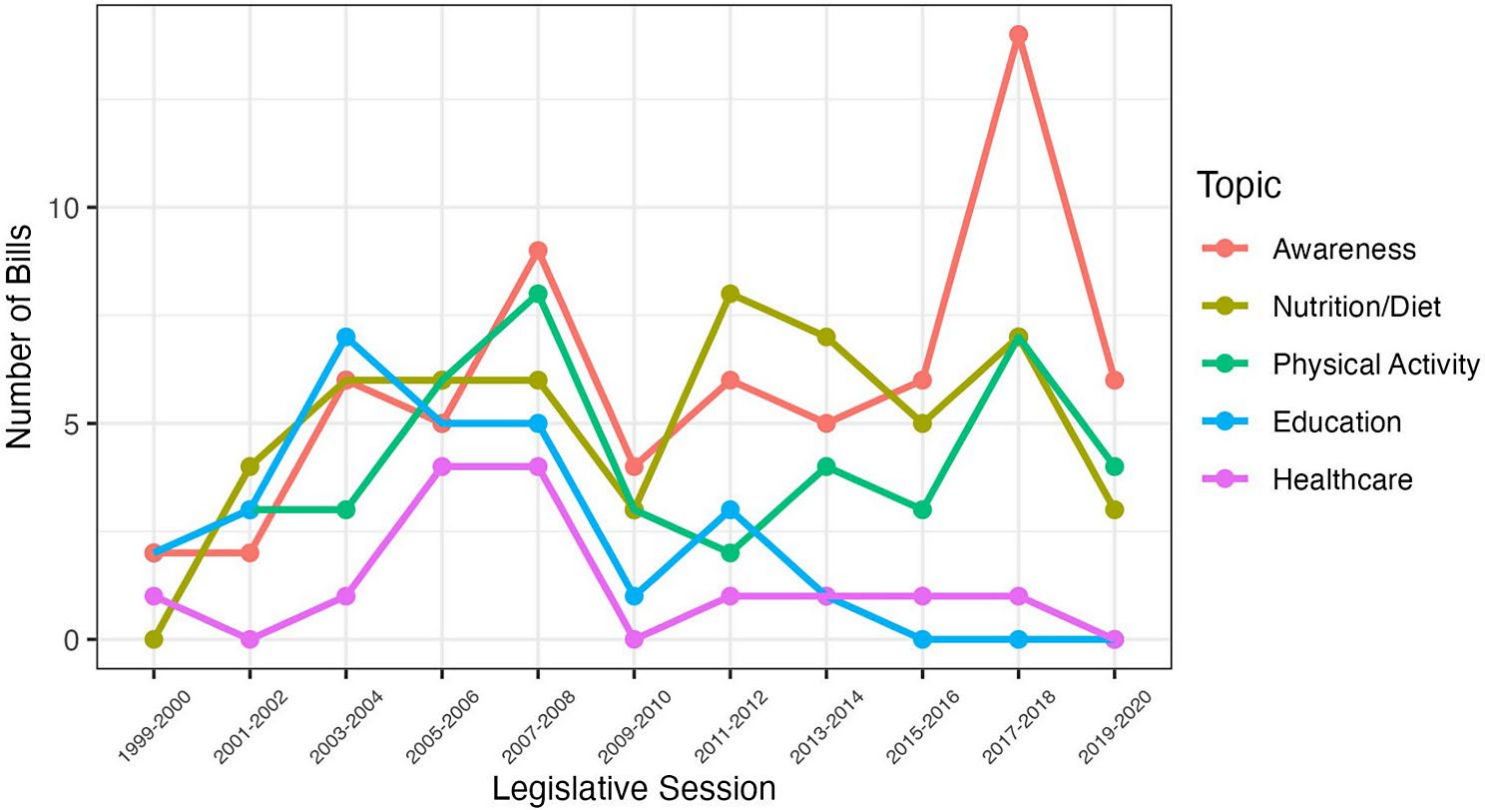
Number of bills and resolutions enacted by policy topic per legislative session in California (N=284), 1999–2020

Figure 1 provides trend lines of enacted bills and resolutions for the five primary topics over time. All five categories experienced increases between 1999 and 2008. In 2009–2010, fewer obesity bills and resolutions were enacted or adopted for each topic. In 2017–2018, awareness bills and resolutions had the highest yearly counts with fourteen, and all of these were resolutions.

A logistic regression predicting enactment included awareness, environmental health, and legislative session found only awareness to be significant. Legislation to raise awareness about obesity had 5.4 times the odds of being passed compared to other policy topics (*p* < 0.001). As seen in Table 2, the rate of enactment is significantly larger in awareness-focused bills and resolutions. This difference was not detected in the sensitivity analysis (results available in Table S4), suggesting resolutions may have driven the effect.

**Table 2:** Results from logistic regression predicting legislative enactment of bills and resolutions to address/prevent obesity in California (*N* = 284), 1999–2020^1^

Coefficients	OR (95% CI)
**Policy Topic: Environmental health**	
Not Included (ref)	---
Included	0.39 (0.08, 1.78)
**Policy Topic: Awareness**	
Not Included (ref)	---
Included	5.39 (2.05, 14.15)***
**Origin**	
Assembly	---
Senate	1.00 (0.99, 1.00)

^1^Standard errors clustered by origin

* *p* < 0.05, ** *p* < 0.01, *** *p* < 0.001

## Discussion

This longitudinal policy surveillance study is the first to examine trends of obesity-related legislation in California over a two-decade period. Between 1999 and 2020, 284 bills and resolutions were introduced in California’s legislature to address obesity. Overall, 24.9% of bills were enacted while 82.1% of resolutions were adopted. At the onset of the time period, California legislators introduced an increasingly higher quantity of bills and resolutions that mentioned obesity, with a peak in 2005–2006 –signaling greater awareness of and interest in this public health issue among state policy stakeholders.

The most common policy topics were nutrition (45% of bills) or physical activity (33%) for introduced legislation. Children and school settings were the most frequently mentioned when a target population or setting was stated, which may be positive since there is evidence regarding the cost-effectiveness of comprehensive school-based interventions to improve dietary behavior and promote physical activity among children [32]. Findings suggest state legislators may be more amenable to introducing paternalistic policy targeting dependents/minors or with greater potential for preventive impact. Public school obesity reduction measures have also been shown to garner greater support among state legislators compared to community-based and tax-related measures [33]. This suggests specific policy topics and instruments might influence legislators’ policy support. For example, taxes may be less likely to be supported as a policy instrument [12, 15]. In California, a statewide sugar-sweetened beverage tax proposal has failed multiple times after obtaining success among some local jurisdictions even with demonstrated evidence of its effectiveness [34]. It is unclear whether evidence regarding the effectiveness of these policies contributes to the success of legislation in the policy process, and this is an important line of inquiry for future research.

Legislation focused on raising obesity awareness had a higher success rate, which was a statistically significant result. This finding may have been driven by the inclusion of resolutions as indicated by results from the sensitivity analysis. Resolutions have been excluded in prior similar work [8] and critiqued as being more ceremonial compared to bills [10]. However, it is important to examine resolutions as well as bills since they represent time and effort expended in the legislative process. Further, our analysis sheds light on an important finding—namely, that awareness promoting legislation may be a more frequently used approach by legislators. A recent study of state legislation to eliminate racial and ethnic health disparities similarly found the most successful bills focused on recognizing or increasing awareness of disparities [35]. These findings call into question as to whether awareness legislation is a meaningful strategy to transform or incrementally advance population health. It is possible that awareness legislation may be a popular policy lever because it is a public signal that legislators are committed to a specific issue without financial or political investment (or a considerable change to the status quo). Future research should examine whether and how awareness-focused legislation might contribute to or influence other programs or activities that impact health.

Most bills and resolutions in the database did not mention a specific target population or setting—only 15% mentioned a specific racial/ethnic group, a missed opportunity to target health inequalities. Identifying effective policies for populations with disproportionately high obesity prevalence rates, like Latinos, African Americans, and low-income households, is particularly crucial. While California’s overall adult obesity prevalence rate in 2022 was 28.1%—lower than most U.S. states—this rate significantly increased during the study period and drastic racial/ethnic health disparities persist. Presently, the adult obesity rate for Hispanic/Latinos is 37.8% and 42.5% among Blacks compared to 25.8% among non-Hispanic whites and 11.1% among non-Hispanic Asians in California [36]. While policy interventions can potentially narrow health disparities [6] by improving conditions, resource availability, or environments in marginalized communities, there are gaps in our understanding of legal approaches and their impact. Additional work should examine potential health equity impacts of proposed and enacted legislation [37] and evaluate how policy instruments might narrow disparities with consideration of potential subgroup differences and the need to tailor policies for subgroups [5].

The database also revealed a decline in the number of introduced obesity legislation focused on healthcare starting in 2009 in California. With the recent advent of glucagon-like peptide 1 receptor agonists (GLP-1 RAs) shown to be associated with dramatic weight loss effects coupled with rapid increased use of these products among patients with obesity [38], we might anticipate an increase in obesity policy focused on healthcare beyond the database period. Future state policy debates may focus on making obesity medication drugs more accessible [39]. States might consider covering these medications as part of their Medicaid program, which may pose budgetary issues given their high costs [40]. Concerningly, policymakers may lose interest in pursuing preventive obesity policy strategies if they prioritize treatment with this class of medications instead of policy to improve the social determinants of health that influence obesity inequities through food-related and physical activity factors [37]. Continuing to monitor obesity policy topic and content trends is an important means to track whether legislators’ commitment to obesity prevention wanes and to assess whether these policies are effective (and for whom) [32].

Results indicate enactment rates were not significantly different by legislative session or originating chamber, suggesting policy success may be limited by more traditional legislative constraints (e.g., time or budget limitations) or views on the role of individual-level behavior or agency contributing to obesity rates [18, 41–43]. Future research should examine political factors that might influence policy success, including legislators’ socio-demographic characteristics, political party affiliation, and ideology since non-white, female, and Democratic policymakers (i.e., Governors and legislators) may be more likely to support obesity reduction legislation [14, 33]. Bipartisan sponsorship and having Republican sponsorship may also improve an obesity bill’s likelihood of enactment [11, 12, 16].

### Strengths and Limitations

Strengths include the methodological approach that accounts for a longer study period than nearly all comparable existing studies. By analyzing data from 20 years with varying epidemiologic trends and political conditions, the resulting database is more comprehensive than others. Next, an advantage to using a legislative website as a data source is that it provides free detailed policy content relative to subscription databases [44]. Other strengths include a comprehensive focus on prevention and reduction mechanisms spanning health behaviors to reflect the spectrum of policy levers used by policymakers. These policies go beyond the health care policy sphere and encompass public health policies that assume a broader perspective of the social and economic determinants of health [18].

Limitations include a lack of a second coder to ensure high interrater agreement and exclusion of bills that may have targeted obesogenic conditions (or contributing factors) but did not mention obesity. Replication elsewhere may be challenging since other state legislative databases may be difficult to access [7, 44]. Further, we focus on state-level legislative policy and did not include executive (gubernatorial) budgetary proposals, case law, regulatory action, or local policy [17]. Notably, California enacts more obesity-related policies than other states, making it an outlier [10–12, 16, 28]. Comparative studies in large states with a different political environment, like Texas, may reveal obesity policy patterns and trends that persist or are contingent on political variables.

## Conclusion

Examining state obesity legislation in California over a twenty-year time span can advance our understanding of historical state health policy trends and elements associated with policy success. This study found California policymakers introduced more legislation focused on children/schools and nutrition and physical activity topics. Results on the success of legislation (and adoption of resolutions) to raise awareness about obesity raises important questions about the effectiveness of legislative policy to reduce and prevent obesity.

## Electronic supplementary material

Below is the link to the electronic supplementary material.

Supplementary Material 1

## Data Availability

The datasets generated and/or analysed during the current study are available in the CHIP Lab website: https://faculty.sites.uci.edu/ddpayan/pubs/data-codebooks/.

## Abbreviations

US: United States
SD: Standard deviation
GLP-1 RAs: Glucagon-like peptide 1 receptor agonists
