# Supplementary material for: Longitudinal policy surveillance of state obesity legislation in California, 1999–2020
Source: BMC Public Health. 2024 Nov 6;24:3064. doi: 10.1186/s12889-024-20557-y (PMC11539810; doi:10.1186/s12889-024-20557-y)
Supplement: Supplementary file 1 — Supplementary Material 1 [file 12889_2024_20557_MOESM1_ESM.doc]

**Systematic Search Process and Additional Analyses (Supplement Material)**

Figure S1: Systematic search process for obesity prevention and reduction bills introduced in California’s legislature (1999-2020)

Bills/resolutions identified from:

California state legislative website (n = 354)

State obesity policy databases* (n = 4)

**Identification**

Bills/resolutions screened

(n = 358)

Bills/resolutions excluded for lack of relevance

(n = 42)

**Screening**

Bills/resolutions sought for retrieval and assessed for eligibility

(n = 316)

Bills/resolutions excluded:

Liability bills (n = 3)

Budget bills (n = 6)

Lacking obesity/ overweight prevention or reduction mechanism (n = 23)

Bills/resolutions included

(n = 284)

**Included**

*Only non-duplicate bills were included from two existing databases

*Table S1. Legislative and policy characteristics of introduced, not enacted, and enacted bills and resolutions to address/prevent obesity in California (N=171), 1999-2020 – Assembly Only*

|  | **Bills Introduced** (N = 171)  n (%) | **Not Enacted**  (N =96)  n (%) | **Enacted** (N =75)  n (%) |
| --- | --- | --- | --- |
| **Legislative Year** |  |  |  |
| 1999-2000 | 3 (1.75) | 1 (1) | 2 (2.7) |
| 2001-2002 | 8 (4.68) | 3 (3.1) | 5 (6.7) |
| 2003-2004 | 11 (6.43) | 4 (4.2) | 7 (9.3) |
| 2005-2006 | 22 (12.87) | 12 (12.5) | 10 (13.3) |
| 2007-2008 | 23 (13.45) | 17 (17.7) | 6 (8) |
| 2009-2010 | 11 (6.43) | 8 (8.3) | 3 (4) |
| 2011-2012 | 23 (13.45) | 14 (14.6) | 9 (12) |
| 2013-2014 | 14 (8.19) | 6 (6.3) | 8 (10.7) |
| 2015-2016 | 14 (8.19) | 9 (9.4) | 5 (6.7) |
| 2017-2018 | 22 (12.87) | 9 (9.4) | 13 (17.3) |
| 2019-2020 | 20 (11.7) | 13 (13.5) | 7 (9.3) |
| **Target Population1** |  |  |  |
| Infants (<2 years of age) | 3 (1.8) | 1 (1.0) | 2 (2.7) |
| Children (2-17 years) | 70 (40.9) | 39 (40.6) | 31 (41.3) |
| Adults (18-64 years) | 3 (1.8) | 2 (2.1) | 1 (1.3) |
| Older adults (65 years+) | 1 (0.6) | 1 (1.0) | 0 (0.0) |
| Other | 0 (0.0) | 0 (0.0) | 0 (0.0) |
| None specified | 94 (55.0) | 53 (55.2) | 41 (54.7) |
| **Race/ethnicity mentioned** | 29 (17.0) | 15 (15.6) | 14 (18.7) |
| **Target Setting** |  |  |  |
| School setting/early childcare facility | 59 (34.5) | 38 (39.6) | 21 (28) |
| Health care organization | 0 (0.0) | 0 (0.0) | 0 (0.0) |
| Restaurant or food facility (excluding school cafeterias) | 2 (1.2) | 1 (1) | 1 (1.3) |
| Employment sites or worksite | 3 (1.8) | 2 (2.1) | 1 (1.3) |
| Other | 7 (4.1) | 3 (3.1) | 4 (5.3) |
| None specified | 100 (58.5) | 52 (54.2) | 48 (64.0) |
| **Policy Topic2** |  |  |  |
| Nutrition/diet | 78 (45.6) | 49 (51) | 29 (38.7) |
| Physical activity | 55 (32.2) | 30 (31.3) | 25 (33.3) |
| Awareness | 53 (31.0) | 12 (12.5) | 41 (54.7)*** |
| Education | 41 (24.0) | 26 (27.1) | 15 (20.0) |
| Health care | 24 (14.0) | 16 (16.7) | 8 (10.7) |
| Environmental health | 9 (5.3) | 8 (8.3) | 1 (1.3)* |
| Abuse | 9 (5.3) | 2 (2.1) | 7 (9.3)* |
| Housing | 2 (1.2) | 2 (2.1) | 0 (0.0) |

1Bill specifies a target age or group (i.e., child/children)

2Topics were not mutually exclusive

*p < 0.05, ** p < 0.01, *** p < 0.001

*Table S2. Legislative and policy characteristics of introduced, not enacted, and enacted bills and resolutions to address/prevent obesity in California (N=113), 1999-2020 – Senate Only*

|  | **Bills Introduced** (N = 113)  n (%) | **Not Enacted**  (N =63)  n (%) | **Enacted** (N =50)  n (%) |
| --- | --- | --- | --- |
| **Legislative Year** |  |  |  |
| 1999-2000 | 4 (3.5) | 3 (4.8) | 1 (2.0) |
| 2001-2002 | 6 (5.3) | 5 (7.9) | 1 (2.0) |
| 2003-2004 | 13 (11.5) | 7 (11.1) | 6 (12.0) |
| 2005-2006 | 21 (18.6) | 12 (19.1) | 9 (18.0) |
| 2007-2008 | 15 (13.3) | 5 (7.9) | 10 (20.0) |
| 2009-2010 | 8 (7.1) | 4 (6.4) | 4 (8.0) |
| 2011-2012 | 11 (9.7) | 5 (7.9) | 6 (12.0) |
| 2013-2014 | 11 (9.7) | 6 (9.5) | 5 (10.0) |
| 2015-2016 | 8 (7.1) | 5 (7.9) | 3 (6.0) |
| 2017-2018 | 7 (6.2) | 3 (4.8) | 4 (8.0) |
| 2019-2020 | 9 (8) | 8 (12.7) | 1 (2.0) |
| **Target Population** |  |  |  |
| Infants (<2 years of age) | 5 (4.4) | 2 (3.2) | 3 (6.0) |
| Children (2-17 years) | 31 (27.4) | 17 (27) | 14 (28.0) |
| Adults (18-64 years) | 2 (1.8) | 2 (3.2) | 0 (0.0) |
| Older adults (65 years+) | 0 (0.0) | 0 (0.0) | 0 (0.0) |
| Other | 3 (2.7) | 2 (3.2) | 1 (2) |
| None specified | 72 (63.7) | 40 (63.5) | 32 (64.0) |
| **Race/ethnicity mentioned** | 13 (11.5) | 9 (14.3) | 4 (8.0) |
| **Target Setting** |  |  |  |
| School setting/early childcare facility | 23 (20.4) | 16 (25.4) | 7 (14.0) |
| Health care organization | 8 (7.1) | 4 (6.4) | 4 (8.0) |
| Restaurant or food facility (excluding school cafeterias) | 3 (2.7) | 1 (1.6) | 2 (4.0) |
| Employment sites or worksite | 2 (1.8) | 2 (3.2) | 0 (0.0) |
| Other | 6 (5.3) | 5 (7.9) | 1 (2.0) |
| None specified | 71 (62.8) | 35 (55.6) | 36 (72.0) |
| **Policy Topic** |  |  |  |
| Nutrition/diet | 51 (45.1) | 25 (39.7) | 26 (52.0) |
| Physical activity | 38 (33.6) | 18 (28.6) | 20 (40.0) |
| Awareness | 38 (33.6) | 14 (22.2) | 24 (48.0) |
| Education | 32 (28.3) | 20 (31.8) | 12 (24) |
| Health care | 20 (17.7) | 14 (22.2) | 6 (12.0) |
| Environmental health | 10 (8.9) | 7 (11.1) | 3 (6.0) |
| Abuse | 2 (1.8) | 2 (3.2) | 0 (0.0) |
| Housing | 1 (0.9) | 1 (0.9) | 0 (0.0) |

1Bill specifies a target age or group (i.e., child/children)

2Topics were not mutually exclusive

*p < 0.05, ** p < 0.01, *** p < 0.001

Table S3. P-values from chi-square tests comparing covariate with bill enactment for full dataset and restricted to only bills

|  | **Total Sample**  **N = 284** | **Only Bills**  **N = 189** |
| --- | --- | --- |
| **Legislative Chamber - Origination** | 0.949 | 0.523 |
| **Legislative Year** | 0.597 | 0.306 |
| **Target Population** | 0.667 | 0.801 |
| **Race/ethnicity mentioned** | 0.870 | 0.478 |
| **Target setting** | 0.219 | 0.140 |
| **Policy Topic** |  |  |
| Nutrition/diet | 0.669 | 0.118 |
| Physical activity | 0.300 | 0.108 |
| Awareness | ***< 0.001*** | 0.894 |
| Education | 0.161 | 0.765 |
| Health care | 0.076 | 0.900 |
| Environmental health | ***0.037*** | 0.757 |
| Abuse | 0.181 | 0.315 |
| Housing | 0.123 | 0.315 |

Table S4. Results from logistic regression predicting legislative enactment of bills and resolutions to address/prevent obesity in California (N = 189), 1999-20201 ­*– restricted to bills only*

| Coefficients | OR (95% CI) |
| --- | --- |
| Policy Topic: Environmental health |  |
| Not Included (ref) | --- |
| Included | 0.79 (0.42, 1.49) |
| Policy Topic: Awareness |  |
| Not Included (ref) | --- |
| Included | 0.9 (0.82, 0.99)* |
| Origin |  |
| Assembly | --- |
| Senate | 1.26 (1.24, 1.28)*** |

1Standard errors clustered by origin

* p < 0.05, ** p < 0.01, *** p < 0.001
